# The complete chloroplast genome sequence of *Abies beshanzuensis*, a highly endangered fir species from south China

**DOI:** 10.1080/23802359.2018.1502638

**Published:** 2018-08-23

**Authors:** Yi-Zhen Shao, Jin-Tao Hu, Peng-Zhen Fan, Yan-Yan Liu, Yi-Han Wang

**Affiliations:** a College of Life Sciences, Henan Agriculture University, Zhengzhou, China;; b College of Plant Protection, Henan Agriculture University, Zhengzhou, China

**Keywords:** Abies beshanzuensis, Chloroplast genome, Endangered, Phylogenetic

## Abstract

*Abies beshanzuensis* is critically endangered and endemic to Zhengjiang province of China, with only three surviving individuals. In present study, we reported the complete chloroplast (cp) genome of *Abies beshanzuensis*. The complete chloroplast genome size is 121,399 bp. In total, 114genes were identified, including 68 peptide-encoding genes, 35 tRNA genes, four rRNA genes, six open reading frames and one pseudogene. Loss of ndh genes was also identified in the genome of *A*. *beshanzuensis* like other genomes in the family Pinaceae. Thirteen genes contain one (11 genes) or two (rps12 and ycf3 genes) introns. Inverted repeat sequences located in 42-kb inversion points (1186 bp) include trnS-psaM-ycf12-trnG genes. In phylogenetic analysis, the tree confirms that the four *Abies* species are strongly supported as monophyletic. The complete plastome of *A*. *beshanzuensis* will provide potential genetic resources for further conservation and evolutionary studies of this highly endangered species.

*Abies* is the second largest genus of family Pinaceae (after *Pinus*), comprising about 50 species (Liu 1971; Farjon 2001). Due to extensive habitat loss by climate change and human activities, many economically and ecologically valuable firs are listed as Threatened or Endangered (Xiang 2015). *Abies beshanzuensis* M.H. Wu is one of the highly endangered firs species, with only three surviving individuals endemic to Mt. Baishanzu of China. Here, we assembled and characterized the complete plastome of *A*. *beshanzuensis*. It will provide potential genetic resources for further conservation and evolutionary studies of *A*. *beshanzuensis*.

The plant material of *Abies beshanzuensis* was collected from a single individual that lives in the natural forest habitat of Mt. Baishanzu in Zhejiang, China. Voucher specimen and DNA sample (Wang X.-Q. 04039) were deposited in the herbarium of Institute of Botany, CAS (PE). In total, ca. 10.4 million high-quality clean reads (150 bp PE read length) were generated with adaptors trimmed. The CLC de novo assembler (CLC Bio, Aarhus, Denmark), BLAST, GeSeq (Tillich et al. 2017), and tRNAscan-SE v1.3.1 (Santa Cruz, USA) (Schattner et al. 2005) were used to align, assemble, and annotate the plastome.

The full length of *Abies beshanzuensis* chloroplast genome (GenBank Accession No. MH476330) was 121,399 bp and comprised of a large single copy region (LSC with 66,657 bp), a small single copy region (SSC with 54,214 bp), and two inverted repeat regions (IR with 264 bp). The overall GC content of the *A*. *beshanzuensis* cp genome was 38.3% and the GC content in the LSC, SSC, and IR regions are 37.4, 39.3, and 39.0%, respectively. A total of 114 genes were contained in the cp genome (68 peptide-encoding genes, 35 tRNA genes, four rRNA genes, six open reading frames and one pseudogene). Fifty-three protein coding, 16 tRNA genes, three open reading frames and one pseudogene are located in the LSC region, while 15 protein-coding, 17 tRNA genes, 4 rRNA and 3 open reading frames are located in the SSC region, respectively. Only one tRNA gene (trnI-CAU) is duplicated and located on the IR regions. All ndh genes have been lost in the genome of *A*. *beshanzuensis* like other cp genomes of family Pinaceae. Among the protein-coding genes, two genes (rps12 and ycf3) contained two introns, and other eleven genes (trnK-UUU, trnV-UAC, rpoC1, atpF, trnG-GCC, petB, petD, rpl16, rpl2, trnL-UAA, trnA-UGC) had one intron each. In previous studies, short inverted repeat sequences which consist of trnS-psaM-ycf12-trnG and trnG-ycf12-psaM-trnS (1186 bp) are located in the cp genome of *A*. *beshanzuensis*. Length and sequence of inverted repeats from *A*. *beshanzuensi* is identical with those of *A. koreana* (Yi et al. 2015). Twelve chloroplast genomes of Pinaceae and Ginkgoaceae were fully aligned with MAFFT v7.3 (Suita, Osaka, Japan) (Katoh and Standley 2013), and the maximum likelihood (ML) inference was performed using GTRþIþC model with RAxML v.8.2.1 (Karlsruhe, Germany) (Stamatakis 2014) on the CIPRES cluster service (Miller et al. 2010). The result revealed that the four *Abies* species (*A. beshanzuensis*, *A. koreana*, *A. neprolepis* and *A. sbirica*) are strongly supported as monophyletic (Figure 1).

**Figure 1.**
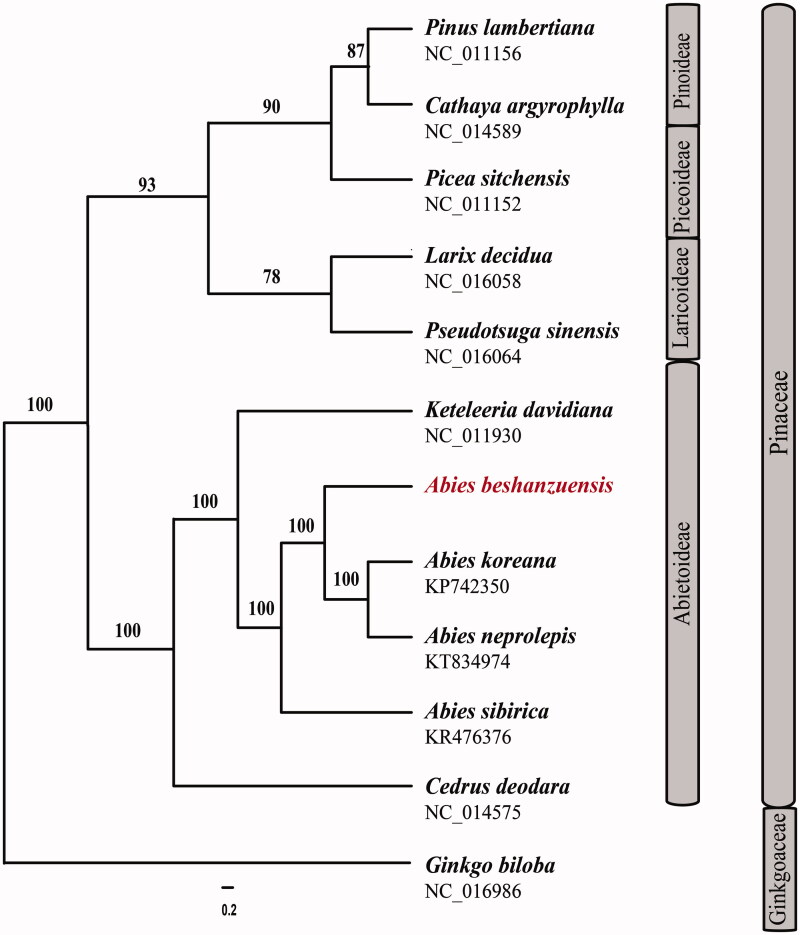
The best Maximum likelihood (ML) phylogram inferred from 12 chloroplast genomes in Pinaceae and Ginkgoaceae (bootstrap value are indicated on the branches).
